# Cytoskeletal Linker Protein Dystonin Is Not Critical to Terminal Oligodendrocyte Differentiation or CNS Myelination

**DOI:** 10.1371/journal.pone.0149201

**Published:** 2016-02-17

**Authors:** Samantha F. Kornfeld, Anisha Lynch-Godrei, Sawyer R. Bonin, Sabrina Gibeault, Yves De Repentigny, Rashmi Kothary

**Affiliations:** 1 Regenerative Medicine Program, Ottawa Hospital Research Institute, Ottawa, Ontario, Canada K1H 8L6; 2 Department of Cellular and Molecular Medicine, University of Ottawa, Ottawa, Ontario, Canada K1H 8M5; 3 Department of Medicine, University of Ottawa, Ottawa, Ontario, Canada K1H 8M5; 4 University of Ottawa Centre for Neuromuscular Disease, Ottawa, Ontario, Canada K1H 8M5; Instituto Cajal-CSIC, SPAIN

## Abstract

Oligodendrocyte differentiation and central nervous system myelination require massive reorganization of the oligodendrocyte cytoskeleton. Loss of specific actin- and tubulin-organizing factors can lead to impaired morphological and/or molecular differentiation of oligodendrocytes, resulting in a subsequent loss of myelination. Dystonin is a cytoskeletal linker protein with both actin- and tubulin-binding domains. Loss of function of this protein results in a sensory neuropathy called Hereditary Sensory Autonomic Neuropathy VI in humans and *dystonia musculorum* in mice. This disease presents with severe ataxia, dystonic muscle and is ultimately fatal early in life. While loss of the neuronal isoforms of dystonin primarily leads to sensory neuron degeneration, it has also been shown that peripheral myelination is compromised due to intrinsic Schwann cell differentiation abnormalities. The role of this cytoskeletal linker in oligodendrocytes, however, remains unclear. We sought to determine the effects of the loss of neuronal dystonin on oligodendrocyte differentiation and central myelination. To address this, primary oligodendrocytes were isolated from a severe model of *dystonia musculorum*, *Dst*^*dt-27J*^, and assessed for morphological and molecular differentiation capacity. No defects could be discerned in the differentiation of *Dst*^*dt-27J*^ oligodendrocytes relative to oligodendrocytes from wild-type littermates. Survival was also compared between *Dst*^*dt-27J*^ and wild-type oligodendrocytes, revealing no significant difference. Using a recently developed migration assay, we further analysed the ability of primary oligodendrocyte progenitor cell motility, and found that *Dst*^*dt-27J*^ oligodendrocyte progenitor cells were able to migrate normally. Finally, *in vivo* analysis of oligodendrocyte myelination was done in phenotype-stage optic nerve, cerebral cortex and spinal cord. The density of myelinated axons and g-ratios of *Dst*^*dt-27J*^ optic nerves was normal, as was myelin basic protein expression in both cerebral cortex and spinal cord. Together these data suggest that, unlike Schwann cells, oligodendrocytes do not have an intrinsic requirement for neuronal dystonin for differentiation and myelination.

## Introduction

Oligodendrocytes (OLs), the myelinating cells of the central nervous system (CNS), undergo complex morphological and molecular changes during differentiation and myelination. As oligodendrocyte progenitor cells (OPCs), they are migratory, mitotic cells with a relatively simple morphology. Upon interaction with appropriate environmental signals, OPCs exit the cell cycle and initiate differentiation to become increasingly branched, non-migratory, post-mitotic OLs. OL branches are able to contact and wrap their membrane around axons to produce the compact myelin necessary for saltatory conduction and trophic and metabolic axonal support [1–3]. Contrary to the myelinating Schwann cells of the peripheral nervous system (PNS), which myelinate axons in a 1:1 ratio, a single oligodendrocyte can contact and myelinate many axons simultaneously [4,5].

Requirements for the transition from OPC to OL are numerous and include both cell intrinsic and extrinsic factors. A major outcome of the initiation of differentiation is massive reorganization of the cytoskeleton into a progressively ramified arrangement. In OLs, this means promoting and maintaining the organization of microfilaments and microtubules, as intermediate filaments are absent from these cells [6,7]. Initial membrane protrusions are instigated by microfilament growth to form filopodia, which then expand to form thicker, microfilament- and microtubule-rich lamellipodia [7]. Multiple actin- and tubulin-organizing factors are necessary and essential to OL differentiation. A loss of OL morphological complexity is observed in the absence of actin-organizing factors Wiscott-Aldrich syndrome protein (N-WASP), WASP family verprolin-homologous protein 1 (WAVE1), focal adhesion kinase (FAK) and integrin-linked kinase (ILK) [8–11], as well as tubulin-organizing factors tubulin polymerization promoting protein (TPPP/p25), 2',3'-cyclic-nucleotide 3'-phosphodiesterase (CNPase), Fyn kinase and tau [12–14], amongst others. As morphological differentiation is a requirement for successful myelination to occur, it is clear that the capacity of an OL for cytoskeletal reorganization is critical to CNS function and health.

One cytoskeleton-organizing factor with an unclear role in OLs is the large cytoskeletal linker protein dystonin (DST), which is a member of the plakin family [15]. Tissue-specific isoforms of Dst are found in neural, muscle and epithelial compartments [16–19]. *Dst* in neural tissue can be alternatively spliced to give rise to the three neuronal transcripts, *Dst-A1*, *-A2* and *-A3*. The proteins encoded by these transcripts differ only in their N-terminal regions which localize them regionally within the cell [15]. In common between the neuronal isoforms are both an actin-binding and a microtubule-binding domain, conferring their function as cytoskeletal linkers [17,20]. Loss of dystonin results in ultimately fatal peripheral neuropathies, namely hereditary sensory and autonomic neuropathy type VI (HSAN-VI) in humans and *dystonia musculorum* (*Dst*^*dt*^) in mice, characterized by ataxia, dystonic muscle and sensory neuron degeneration [21,22].

While pathology of sensory neurons is the major contributor to *Dst*^*dt*^ pathogenesis, investigation has also been undertaken to assess the effects of neuronal Dst loss in myelinating glial cells. Two models of *Dst*^*dt*^, *Dst*^*dt-Tg4*^ (lacking Dst-A1 and -A2) and *Dst*^*dt-27J*^ (lacking all neuronal isoforms), have peripheral myelination abnormalities that were attributable to intrinsic differentiation defects in Schwann cells [23]. Subsequently, CNS myelination was explored in *Dst*^*dt-Tg4*^ animals through analysis of optic nerve and spinal cord, and it was suggested that OLs are unable to myelinate normally in the absence of Dst [24]; however, tools for comprehensive analysis of the character of these OLs were lacking at the time, leaving conclusions about the intrinsic role of neuronal Dst in OL differentiation and myelination somewhat ambiguous.

Here, we take advantage of a recently developed method for primary culture of mouse OLs [25], as well as an assay to measure OL migratory capacity [26] to characterize the morphological and molecular differentiation of OLs from the more severe *Dst*^*dt-27J*^ model of *Dst*^*dt*^, which lacks all neuronal DST isoforms. While we show that both OPCs and OLs do express all three neuronal isoforms of *Dst* endogenously, OLs isolated from *Dst*^*dt-27J*^ animals do not show a deficiency in morphological or molecular maturity *in vitro*. Survival is not compromised in OLs lacking Dst, and OPC migration is unaffected. Further, contrary to previous observations made in the less severe *Dst*^*dt-Tg4*^ model, we found no defects in myelin sheath thickness or MBP expression *in vivo*. Taken together, our *in vitro* and *in vivo* findings suggest that OLs do not possess a critical requirement for Dst in differentiation and myelination.

## Materials and Methods

### Animals

For qRT-PCR experiments, wild-type Sprague-Dawley rats were obtained from Charles River. The mutant mouse line *Dst*^*dt-27J*^ was used for all other experiments. This line arose from a spontaneous mutation in the *Dst* allele, which confers phenotype in a recessive manner. The mutant animals were first identified at the Jackson Laboratory, and were then characterized as expressing very low levels of neuronal *Dst* transcript levels relative to wild-type animals [27]. The animals were sacrificed at P0-P1 for primary OL cell culture and at P15 for *in vivo* analyses. Genotypes were determined by PCR amplification of genomic tail DNA. The University of Ottawa Animal Care Committee approved all experimental protocols. The protocols conformed to or exceeded those defined in the Canadian Council on Animal Care's Guide to the Care and Use of Experimental Animals, and the Animals for Research Act.

### Cell culture

Primary rat OPC and OL cultures were generated from P2 cerebral cortex tissue as described previously [28]. In brief, cortices were dissociated with 0.01% trypsin (Sigma) in the presence of 10 μg /mL DNase (Sigma). Mixed glial cultures were plated in DMEM (Wisent) containing 20% fetal bovine serum, 2% Glutamax (Gibco) and 0.5% penicillin/streptomycin (Gibco) on poly-L-lysine-coated filter cap flasks for 10 days at 37°C and 5% CO_2_. Flasks were then shaken overnight at 220 rpm, and medium containing suspended OPCs was removed from the flasks. Contaminating glial cells were removed from suspension by differential adhesion. To obtain proliferating OPC cultures, OPCs were plated in SATO medium with 2% Glutamax, 0.1% bovine serum albumin (Sigma), 50 μg/mL apo-transferrin (Sigma), 5 μg/mL insulin (Sigma), 30 nM sodium selenite (Sigma), 10 nM D-biotin (Sigma) and 10 nM hydrocortisone (Sigma) supplemented with 10 ng/mL PDGF-AA (Millipore) and 10 ng/mL bFGF (Millipore). To obtain differentiating OL cultures, OPCs were plated in SATO medium as above but supplemented with 5 μg/mL N-acetyl-L-cysteine (Sigma), 15 nM triiodothyronine (Sigma) and 10 ng/mL ciliary neurotrophic factor (CNTF; AbD Serotec).

Primary mouse OL cultures were generated from P0 cerebral cortex tissue as described previously [25]. Briefly, cortices were dissociated with papain, and mixed glial cultures were plated in DMEM containing 10% fetal bovine serum, 1% Glutamax, 5 μg/mL insulin and 0.33% penicillin/streptomycin on poly-L-lysine-coated filter cap flasks for 6 days at 37°C and 8.5% CO_2_. Medium was further supplemented with 5 μg/mL insulin and cultures were kept at 5% CO_2_ for an additional 3–4 days. Cultures were then shaken overnight at 220 rpm, and enriched for OPCs by differential adhesion. The OPCs were then plated in differentiating SATO medium with 0.5% fetal bovine serum, 1% Glutamax, 5 μg/mL insulin, 50 μg/mL holo-transferrin (Sigma), 50 ng/mL CNTF and 0.33% penicillin/streptomycin on coverslips dual-coated with poly-L-lysine and laminin and kept at 37°C and 5% CO_2_.

### OPC migration assay

OPC aggregates were collected and plated as described previously [26]. In brief, following shaking of the mixed glial cultures, medium containing suspended OLs was filtered through 0.45 μm mesh. The mesh was then inverted, and a pipet was used to wash medium back through the mesh to dislodge isolated OPC aggregates. These were collected in a dish, and a dissection microscope was used to identify and remove single aggregates to be plated on laminin-coated coverslips. The aggregates were placed in conditioned mixed glial culture medium (containing insulin) collected from flasks prior to shaking. Cells were left to migrate for 4 and 24 hours prior to fixation. For quantification, concentric circles were overlaid placing the original OPC aggregate at the center. Rings were set at 50 μm increments from the aggregate for migration at 4 hours, and at 100 μm increments for 24 hours migration.

### qRT-PCR

RNA was isolated from ~1.5 x 10^5^ primary rat OPCs and OLs using the RNeasy Mini Kit (Qiagen). Samples were collected from proliferating OPCs 4 hours post-seeding in proliferation medium, and from a mixed population of pre-myelinating and myelinating OLs on day 1.5 post-seeding in differentiation medium. Samples were reverse-transcribed with RT^2^ First Strand cDNA synthesis kit (Qiagen) using 120 ng total RNA. Relative expression of each of the three neuronal *Dst* transcripts in proliferating OPCs and differentiation OLs was determined by qRT-PCR. Briefly, each 25 μL reaction contained 5–12 μL total cDNA, 12.5 μL SsoFast EvaGreen Supermix (Bio-Rad), either 0.2 μL 10 μM forward + reverse primers for dystonin-A1, 1 μL 10 μM forward + reverse primers for dystonin-A2 and–A3 transcripts or 0.2 μL 10 μM forward + reverse primers for actin transcripts, and RNase-free water. All samples were amplified using the following protocol: 3 min at 95°C, followed by 40 cycles of 95°C for 10 sec, 60°C for 10 sec and 72°C for 30 sec. Runs were amplified using a Bio-Rad CFX96 and analysed using qBase+ software. For each primer set, 4–6 biological replicates were analysed (n = 5–6, n = 1 animal) and samples were run in technical quadruplicates. Technical replicates were used for analysis only when Cq values differed by 0.25 or less. The following primers were used for amplification: *Dst-A1* forward CTA CAT GTA CGT GGA GGA GCA (779 bp), *Dst-A2* forward GAG GGC TGT GCT TCG GAT AG (741 bp), *Dst-A3* forward GTC TCC AAG GAT GCA CCT AGG GAT, *Dst-A1/A2/A3* reverse CAT CGT TTG CAC CAA TGC C, actin forward CCG TCA GGC AGC TCA TAG CTC TTC, and *β-actin* (*actb)* reverse CTG AAC CCT AAG GCC AAC CGT. Bands for each transcript were visualized by running qRT-PCR products in a 1% agarose gel containing RedSafe^™^ nucleic acid staining solution.

### Immunofluorescence

All samples were fixed in 3% paraformaldehyde. Cells were fixed for 15 min at room temperature, while OPC aggregates were fixed overnight at 4°C. Following fixation, coverslips were washed with PBS, permeabilized for 5 min in 0.1% Triton-X (Sigma), and blocked for 1 hour in 10% goat serum. Cells stained for F-actin were additionally blocked with 0.1% bovine serum albumin (BSA) for 30 min, and incubated with 1:50 rhodamine-phalloidin (Life Technologies) in 0.1% BSA for 45 min at room temperature. Samples were incubated with primary antibody in 10% goat serum overnight at 4°C, at the following concentrations: NG2 1:250 (EMD Millipore), MAG 1:50 (EMD Millipore), MBP 1:100 (AbD Serotec), Olig2 clone 211.F1.1 1:50 (EMD Millipore), and cleaved-caspase 3 (CC3) 1:100 (Cell Signaling). Cells were again washed with PBS, and then incubated with secondary antibodies (Alexa-555, Alexa-488, Alexa-647; Invitrogen) at 1:200 in 10% goat serum for 2 hours at room temperature. All samples were counterstained with Hoechst and mounted in Dako fluorescent mounting medium. For maturation marker and CC3 analyses, n = 3 for both WT and *Dst*^*dt27*^. For quantification of cells undergoing caspase-mediated apoptosis, the proportion of CC3^+^/Olig2^+^ cells out of all Olig2^+^ cells was calculated, to avoid quantification of any contaminating cells. Quantification was done for 20–30 photographs taken at 20X magnification from at least two separate coverslips.

### Morphological analysis

Sholl analysis was used to quantify branching complexity of DD3 OLs by overlaying concentric circles at 30 μm increments from the cell body using ImageJ. Branch intersections were counted at each ring. Cells used for Sholl analysis were co-stained for F-actin to visualize branches and Olig2 to confirm OL identity. For DD6 OLs, total membrane area was measured by tracing total cell outline using ImageJ. Cells used for quantification of membrane area were co-stained with MAG and MBP to visualize both non-compact and compact myelin membrane. For both Sholl and membrane area analyses, n = 3 for WT and *Dst*^*dt-27*^. Sholl analysis was conducted for 30 cells per n, and membrane area was measured for 60 cells per n. Cells were deemed membrane positive when they showed any MBP stained region in which branches were no longer discernible but instead were replaced by flattened membrane sheet of any size. Membrane negative cells showed no MBP staining and no membrane sheets. The presence or absence of membrane was quantified for n = 3 for WT and *Dst*^*dt-27*^. For each n, 20–30 photographs taken at 20X magnification were analysed from at least two separate coverslips.

### Western blotting

Cerebral cortex and spinal cord samples were collected from P15 WT and *Dst*^*dt-27*^ animals and flash-frozen in liquid nitrogen. Tissue from one animal was considered 1 n, and a total of n = 6 was collected. Protein was isolated by gently homogenizing tissue in 1x RIPA lysis buffer (Sigma) on ice. The lysate was centrifuged at 4°C at high speed to remove insoluble material. Samples were separated by SDS-PAGE in a 15% gel. Membranes were incubated in 1:1000 CNPase (Abcam), 1:1000 MOG (Abcam), 1:1000 MBP (AbD Serotec) and 1:50,000 alpha-tubulin (Cell Signaling) primary antibodies overnight at 4°C in Odyssey blocking buffer (Li-Cor Biosciences). Membranes were washed in 1X TBS, then incubated with secondary antibody (IRDye 680RD and 800CW; Li-Cor Biosciences) at 1:10,000 in Odyssey blocking buffer for 1 hour at room temperature. Membranes were visualized and bands quantified using the Li-Cor Odyssey CLx Infrared Imaging System.

### Transmission Electron Microscopy (TEM)

P15 WT and *Dst*^*dt-27J*^ mice were anesthetized by CO_2_ and transcardially perfused with 5 mL of phosphate-buffered saline (PBS) followed by 10 mL of Karnovsky’s fixative (4% paraformaldehyde, 2% glutaraldehyde and 0.1 M sodium cacodylate in phosphate-buffered saline, pH 7.4). Optic nerves were collected and fixed overnight at 4°C in the same fixative. Following fixation, 1 mm segments were cut transversely from the mid-point of each nerve. Segments were subsequently washed twice in 0.1 M sodium cacodylate buffer for 1 hour and again overnight at room temperature. Segments were post-fixed with 1% osmium tetroxide in 0.1 M sodium cacodylate buffer for 1 hour at room temperature. These were washed twice in distilled water for 5 min, and then dehydrated twice for 20 min for each step in a graded series of ethanol from water through 30%-50%-70%-85%-95% ethanol. This was followed by two 30 min washes in 100% ethanol, two 15 min washes in 50% ethanol/50% acetone, and two 15 min washes in 100% acetone. Optic nerve segments were infiltrated in 30% spurr resin/acetone for 20 min and once overnight, then in 50% spurr resin/acetone for 6 hours, and finally in 100% spurr resin overnight. Segments remained in 100% spurr resin, which was changed twice a day for three days at room temperature. All infiltration steps were performed on a rotator. Specimens were embedded in fresh liquid spurr resin and then polymerized overnight at 70°C. Ultrathin sections (80 nm) of the optic nerve segments were collected onto 200-mesh copper grids and dried overnight prior to staining. Ultrathin sections were stained with 2% aqueous uranyl acetate and Reynold’s lead citrate. Sections were observed under a transmission electron microscope (Hitachi 7100).

Optic nerves were collected from n = 4 for both WT and *Dst*^*dt-27J*^. Quantification of myelinated axons was done for 12 electron micrographs per n taken at 4000X. G-ratio was measured by calculating the diameter of the axon/diameter of the axon+myelin using ImageJ. For each n, 25 g-ratios were measured for a total of 100 measurements from both WT and *Dst*^*dt-27J*^.

### Toluidine blue staining

Semithin transverse sections (0.5 μm) of optic nerves were embedded in resin, mounted on glass slides, and stained with toluidine blue. Sections were scanned with a MIRAX MIDI (Zeiss) and viewed using Zeiss MIRAX viewer software. These samples were used for qualitative optic nerve assessment only.

### Statistical analysis

All statistics were done using Prism 6 GraphPad software, with the exception of the qPCR data, which was analysed using qbase+ software (Biogazelle). All two-way comparisons were done using a two-tailed Student’s t-test. Comparisons for qPCR data were done using one-way ANOVA followed by Tukey’s *post hoc* test. Analysis of g-ratios by axon caliber was done by linear regression analysis. Data shown represent mean ± SEM with the exception of myelinated axon density and mean g-ratio, which represent mean ± SD.

## Results

### OPCs and OLs endogenously express all neuronal *Dst* transcripts

**Fig 1:**
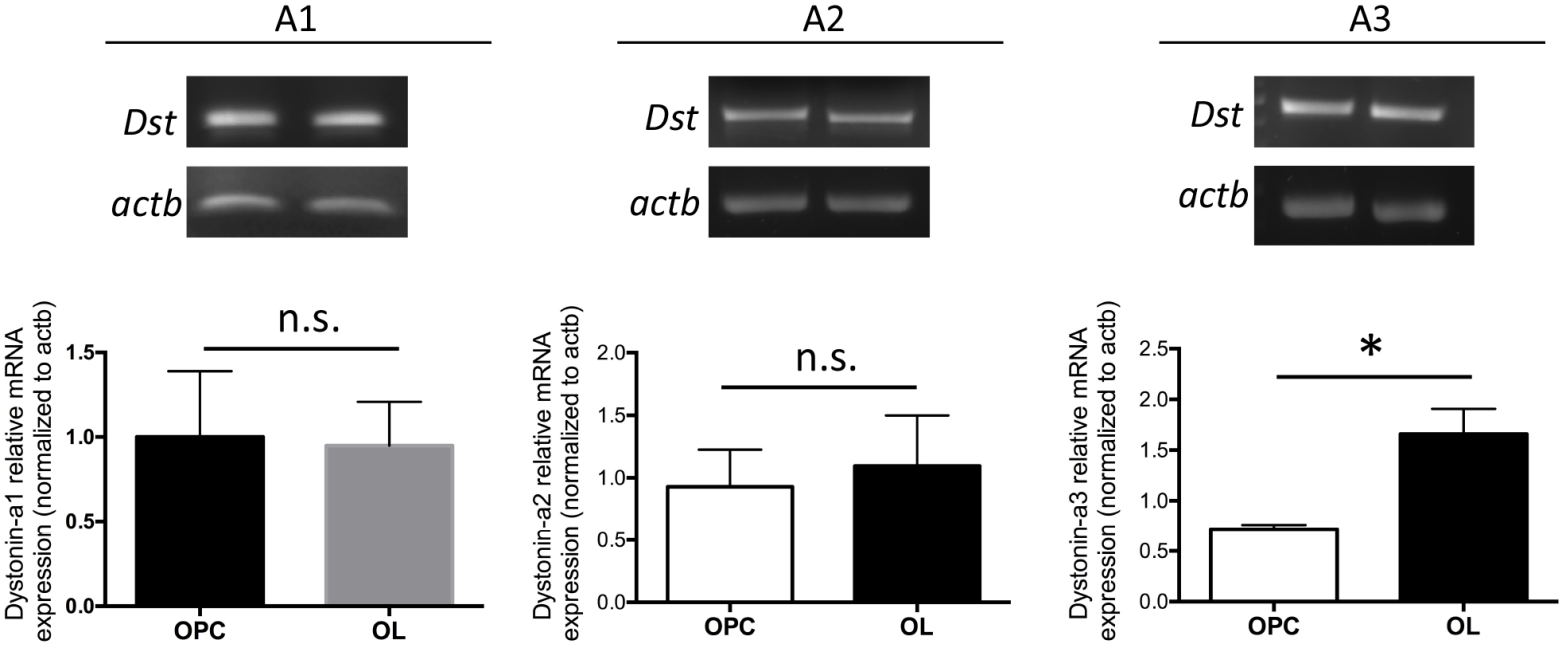
Proliferating OPCs and differentiating OLs express neuronal *Dst* transcripts. Top. Representative RT-PCR products from *Dst*-*A1*, *-A2* and *-A3* with *actb* loading control in primary proliferating OPCs (left lanes) and differentiating OLs (right lanes). Bottom. qRT-PCR analysis of *Dst*-*A1*, *-A2* and *-A3* expression in primary proliferating OPCs and differentiating OLs. n = 4–6; ΔΔCt, *Dst* normalized to *actb*. * p<0.05, n.s. = p≥0.05; two-tailed Student’s t-test. Data represent mean ± SEM.

Expression of neuronal dystonin in CNS tissue has previously been demonstrated by both RNA *in situ* hybridization and RT-PCR [24,29,30]. However, we wished to confirm expression of the three transcripts specifically in OLs. Using RNA extracted from primary proliferating OPCs and differentiating OLs we performed qRT-PCR for *Dst-A1*, *-A2*, and *-A3*. All three transcripts are expressed in proliferating OPCs, and in differentiating OLs (Fig 1). *Dst-A1* and *-A2* showed similar expression levels between OPCs and differentiating OLs, while *Dst*-*A3* showed significantly increased expression in differentiation OLs relative to proliferating OPCs.

### Loss of neuronal Dst does not affect morphological maturation *in vitro*

**Fig 2:**
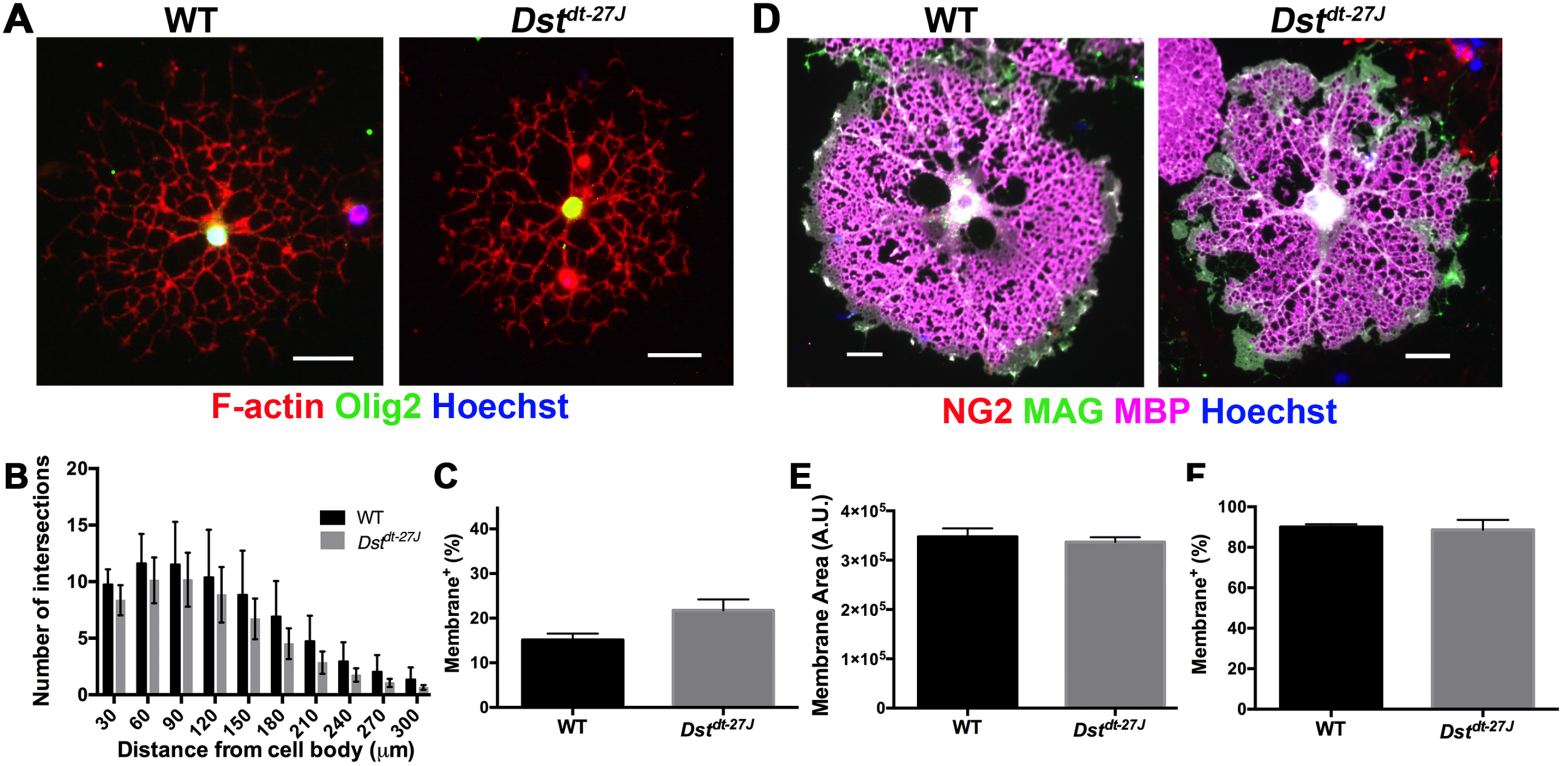
*Dst*^*dt-27J*^ OLs exhibit normal morphological differentiation. A. Immunofluorescence micrographs of WT and *Dst*^*dt-27J*^ showing branching at DD3. B. Sholl analysis of DD3 OLs. Rings were set at 30 μm increments for quantification of intersections. C. Quantification of the percentage of membrane positive OLs at DD3. D. Immunofluorescence micrographs of WT and *Dst*^*dt-27J*^ total membrane production at DD6. E. Quantification of membrane area at DD6. F. Quantification of the percentage of membrane positive OLs at DD6. B, C, E, F: n = 3; all comparisons non-significant by two-tailed Student’s t-test. Data represent mean ± SEM. Scale bars = 50 μm.

It has been illustrated in many instances that loss of cytoskeleton-interacting proteins bears a negative effect on OL differentiation and myelin membrane formation. Since Dst is able to directly interact with both microfilaments and microtubules, we first sought to determine whether loss of dystonin affects the morphological complexity of differentiating OLs. We isolated mixed glial cultures from *Dst*^*dt-27J*^ mice and WT littermates, and then further isolated OPCs to produce purified cultures as described previously [25]. OPCs were allowed to differentiate on a mixed PLL/Ln2 substrate for three days (DD3) or six days (DD6), representing intermediately differentiated or terminally differentiated time points, respectively. At DD3, Sholl analysis was used to assess complexity but revealed no significant differences in branch extension between *Dst*^*dt-27J*^ and WT OLs (Fig 2A and 2B). OLs in the two conditions were also equally capable of producing membrane at this stage of differentiation, as evidenced by similar proportions of membrane-positive cells (Fig 2C). This was maintained at DD6, where again similar proportions of cells were capable of producing membrane (Fig 2F). Cell complexity was further assessed at DD6 by measuring membrane area, which again revealed no differences between *Dst*^*dt-27J*^ and WT OLs (Fig 2D and 2E).

### Loss of neuronal Dst does not affect molecular OL maturation *in vitro*

**Fig 3:**
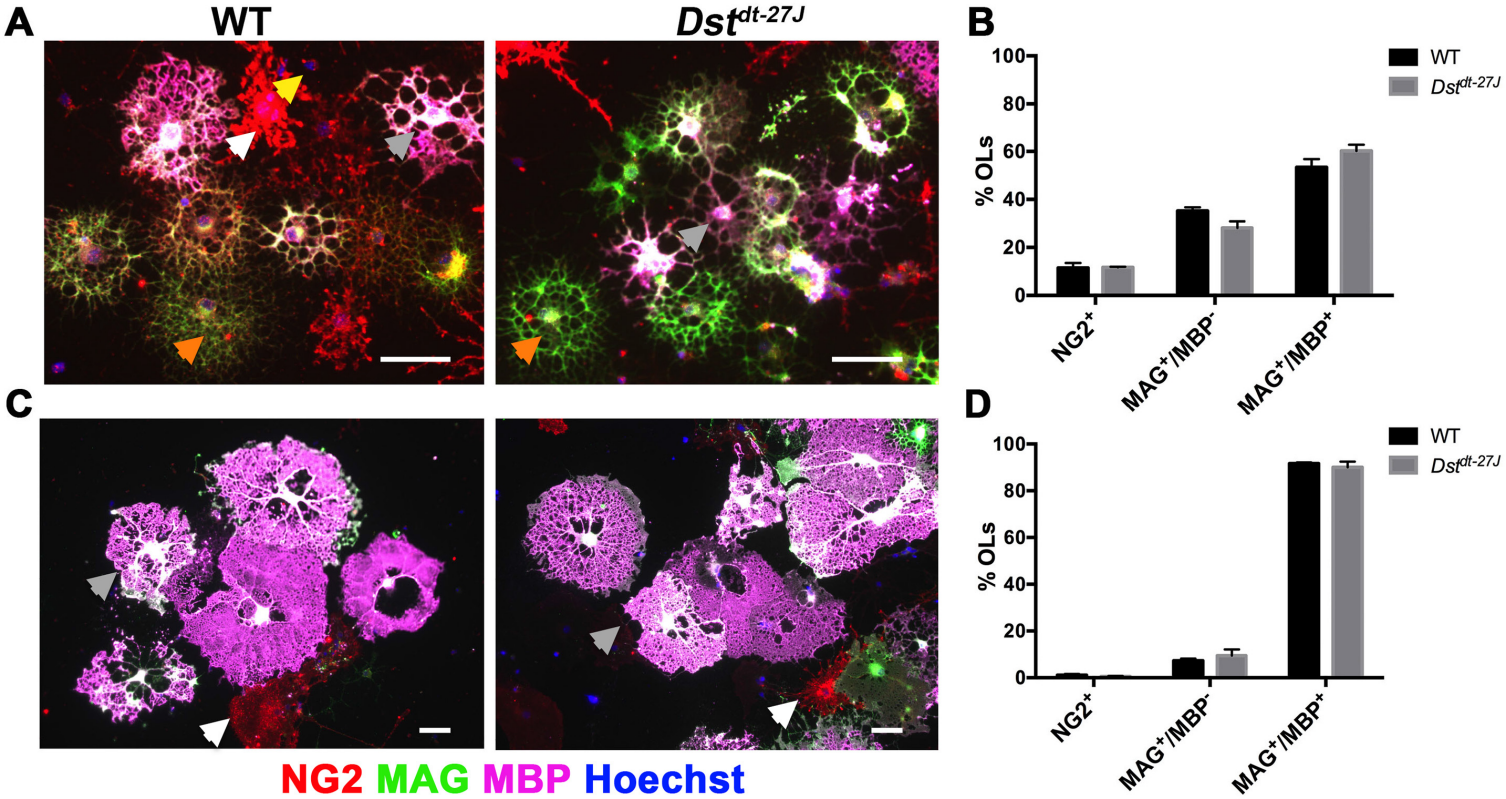
*Dst*^*dt-27J*^ OLs exhibit normal molecular differentiation. A. Immunofluorescence micrographs of WT and *Dst*^*dt-27J*^ showing maturation marker expression at DD3. B. Quantification of the proportion of NG2^+^, MAG^+^/MBP^-^, and MAG^+^/MBP^+^ OLs at DD3. C. Immunofluorescence micrographs of WT and *Dst*^*dt-27J*^ showing maturation marker expression at DD6. D. Quantification of the proportion of NG2^+^, MAG^+^/MBP^-^, and MAG^+^/MBP^+^ OLs at DD6. B, D: n = 3; all comparisons non-significant by two-tailed Student’s t-test. Data represent mean ± SEM. Arrowheads: yellow = NG2^+^, orange = MAG^+^/MBP^-^, grey = MAG^+^/MBP^+^, white = contaminating cell. Scale bars = 50 μm.

While OL morphological maturity is associated with increased branch complexity and eventual membrane formation, molecular maturity involves decreased expression of NG2 concurrent with increased expression first of MAG followed by MBP. To determine whether *Dst*^*dt-27J*^ OLs were able to follow a normal molecular differentiation time course, we assessed proportions of NG2+ (OPC stage), MAG+/MBP- (intermediate OL stage) and MAG+/MBP+ (terminal OL stage) cells at both DD3 and DD6. No significant differences were found in the presence of any of these markers at DD3 (Fig 3A and 3B) or DD6 (Fig 3C and 3D), indicating that OLs lacking DST are able to follow a normal molecular differentiation pattern *in vitro*.

### Apoptosis is not induced in OLs in vitro in the absence of neuronal Dst

**Fig 4:**
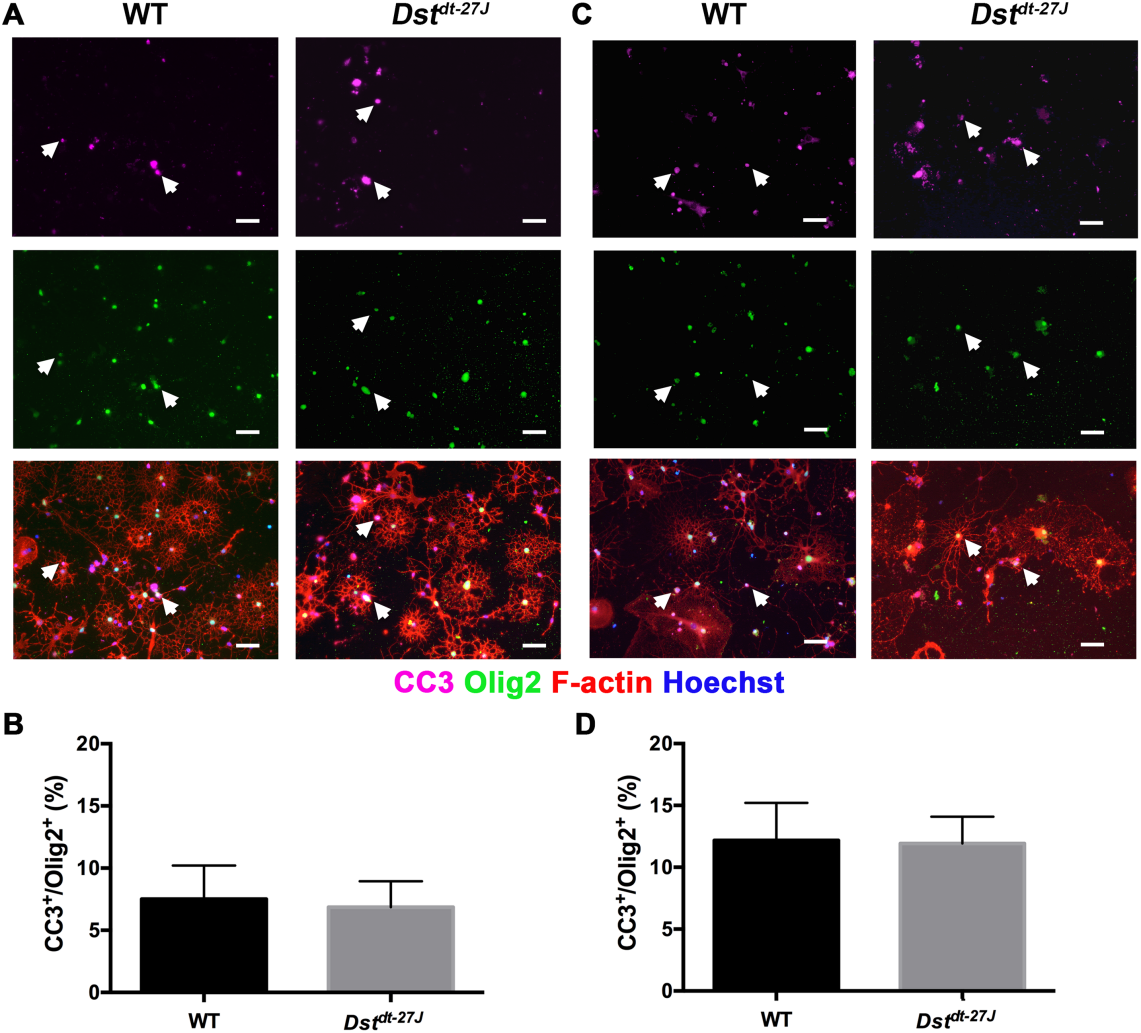
Apoptosis is not increased in *Dst*^*dt-27J*^ OLs. A. Immunofluorescence micrographs of WT and *Dst*^*dt-27J*^ showing colocalization of nuclear CC3 and Olig2 in apoptotic OLs at DD3. B. Quantification of the proportion of CC3^+^/Olig2^+^ relative to total Olig2^+^ OLs at DD3. C. Immunofluorescence micrographs of WT and *Dst*^*dt-27J*^ showing colocalization of nuclear CC3 and Olig2 in apoptotic OLs at DD6. D. Quantification of the proportion of CC3^+^/Olig2^+^ relative to total Olig2^+^ OLs at DD6. A, C: Arrowheads represent CC3^+^/Olig2^+^ OLs. B, D: n = 3; all comparisons non-significant by two-tailed Student’s t-test. Data represent mean ± SEM. Scale bars = 50 μm.

To ensure that the apparent health of Dst-deficient OLs was not isolated to a subpopulation of surviving cells, we assessed OL cultures at both DD3 and DD6 for apoptosis. Differentiating OLs were not undergoing significantly more apoptosis at either DD3 or DD6, as assessed by the presence of apoptotic marker cleaved-caspase 3 (Fig 4).

### OPCs lacking neuronal Dst migrate normally *in vitro*

**Fig 5:**
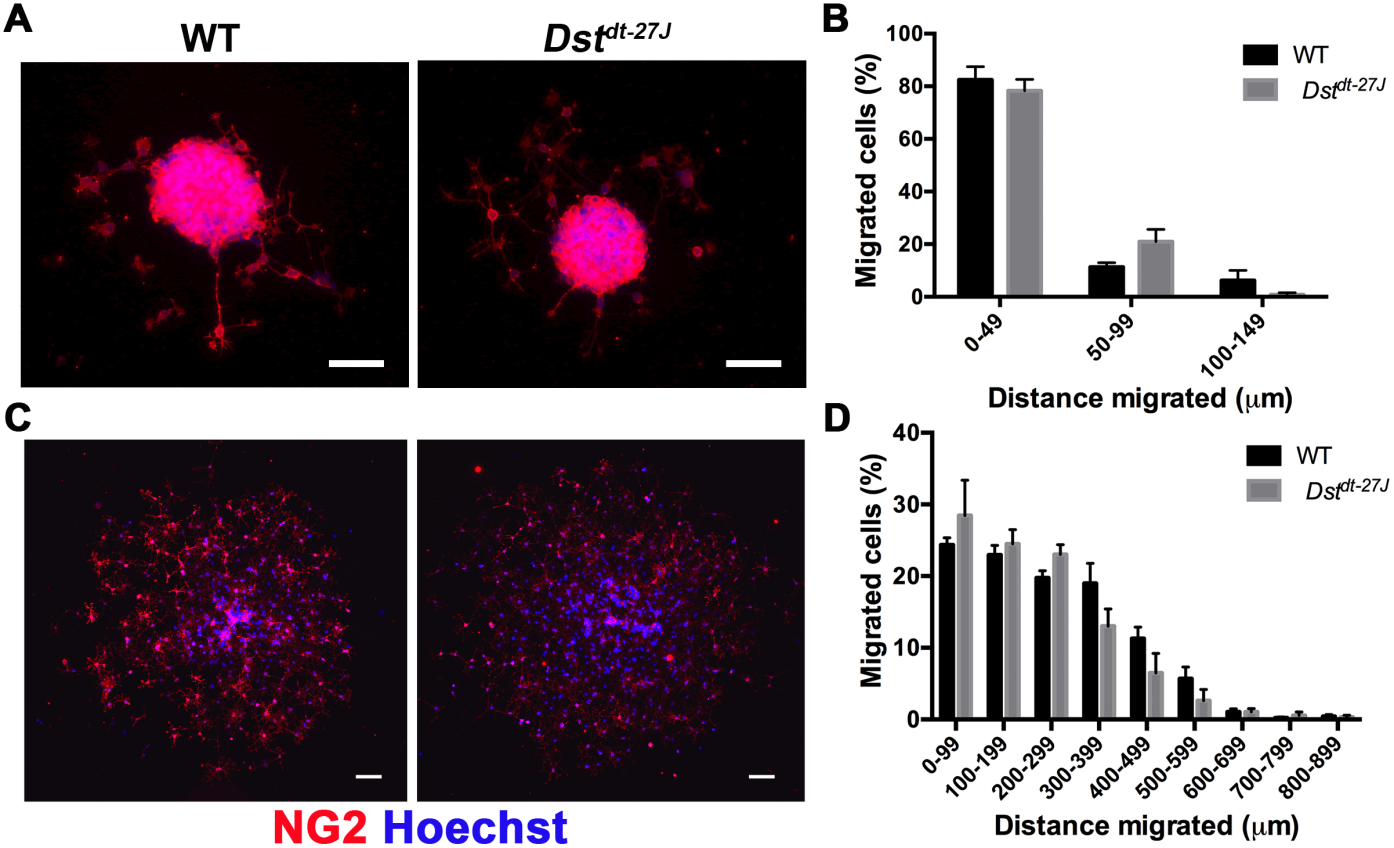
Migration is normal in *Dst*^*dt-27J*^ OPCs. A. Immunofluorescence micrographs of WT and *Dst*^*dt-27J*^ of OPC aggregates 4 hours post-seeding. B. Quantification of the proportion of NG2^+^ OPCs migrated at 4 hours within rings set at 50 μm increments from the aggregate. C. Immunofluorescence micrographs of WT and *Dst*^*dt-27J*^ of OPC aggregate 24 hours post-seeding. D. Quantification of the proportion of NG2^+^ OPCs migrated at 24 hours within rings set at 100 μm increments from the center of the aggregate. B, D: n = 3; all comparisons non-significant by two-tailed Student’s t-test. Data represent mean ± SEM. Scale bars = 50 μm.

While the effects of loss of neuronal Dst have been little investigated, loss of muscle dystonin has been found to impair migration in myoblasts [31]. Since OPCs are also highly migratory cells, we sought to determine if their motility is affected by a lack of neuronal dystonin. Using a recently established method (ROM unpublished) OPC aggregates were isolated from OPCs in suspension and plated on laminin-2 substrate. Aggregates of roughly equal size were selected and allowed to migrate for 4 or 24 hours. Migration was assessed by quantifying the proportion of total NG2^+^ cells found at set distances from the original aggregate after 4 and 24 hours post-seeding. Migration ability was similar between *Dst*^*dt-27J*^ OPCs and WT OPCs at both 4 hours and 24 hours (Fig 5).

### CNS myelination is normal in mice lacking neuronal Dst

*In vitro* analyses revealed no intrinsic differentiation or migration defects in OPCs/OLs lacking DST; however, this does not preclude myelination deficiencies from occurring *in vivo*. Thus, we assessed myelination of the optic nerve in P15 *Dst*^*dt-27J*^ mice along with WT littermates for comparison. In this model, P15 is end-stage phenotype, and all *Dst*^*dt-27J*^ animals exhibited typical *Dst*^*dt*^ symptoms of ataxia, hind-limb clasping and twisting of the trunk [15].

**Fig 6:**
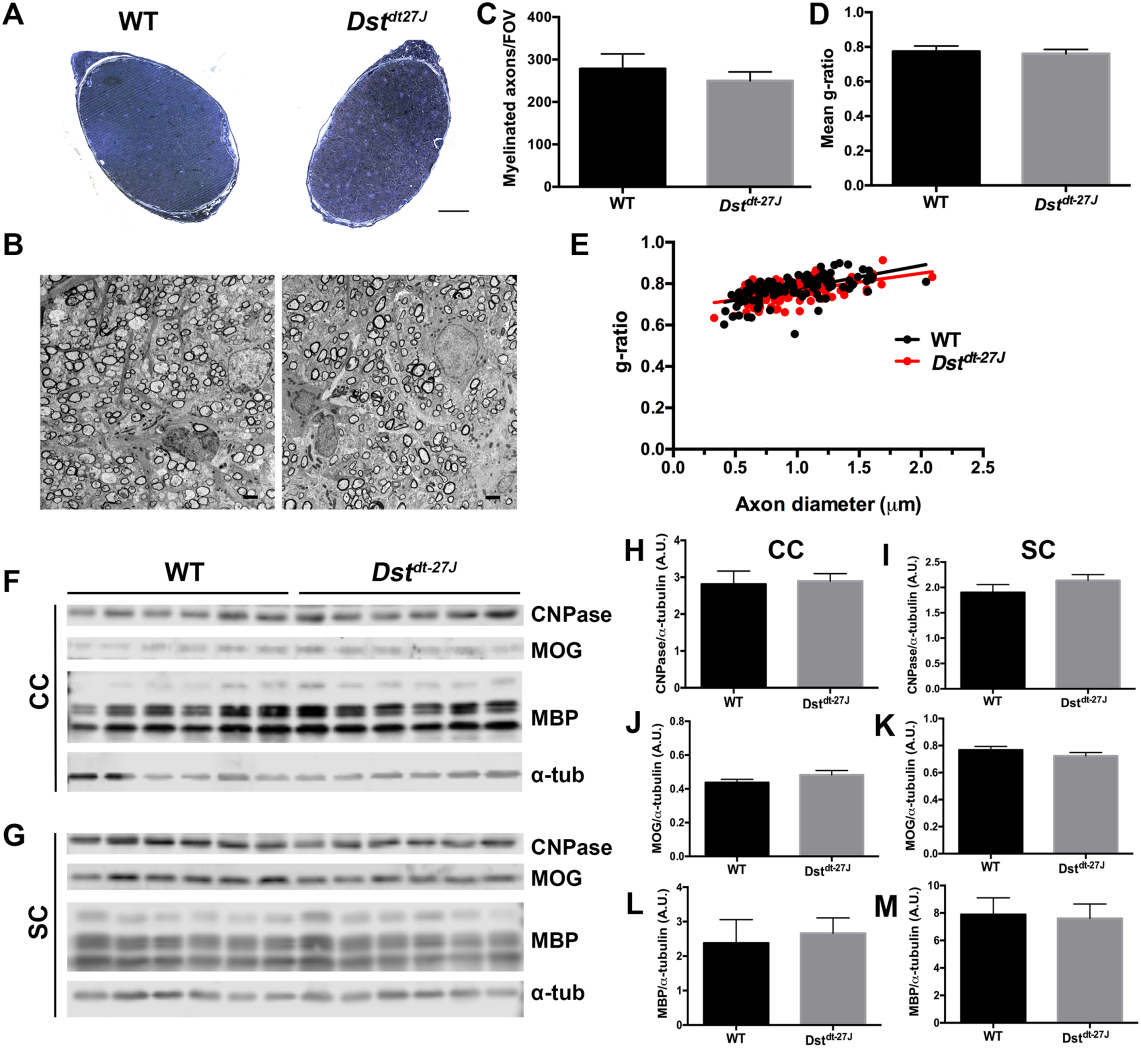
Myelination occurs normally *in vivo* in *Dst*^*dt-27J*^ animals. A. Light microscope images of transected toluidine blue-stained optic nerves from P15 WT and *Dst*^*dt-27J*^ mice. B. Electron micrographs of transected optic nerves from P15 WT and *Dst*^*dt-27J*^ mice. C. Quantification of the number of myelinated axons per field of view (FOV) in optic nerves from P15 WT and *Dst*^*dt-27J*^ mice. D. Quantification of average g-ratio of all myelinated axons per FOV in optic nerves from P15 WT and *Dst*^*dt-27J*^ mice. E. g-ratios plotted by axon caliber in optic nerves from P15 WT and *Dst*^*dt-27J*^ mice. F, G. Fluorescence Western blot analysis of CNPase, MOG, MBP and α-tubulin (α-tub) from cerebral cortex (CC—top panels) and spinal cord (SC—bottom panels) from P15 WT and *Dst*^*dt-27J*^ mice. Protein sizes—CNPase: 48 kDa; MOG: 27 kDa; MBP isoforms, from top to bottom: 21.5 kDa, 18.5 kDa, 17.0 kDa, 14.0 kDa. α-tub: 52 kDa. H, I. Quantification of total CNPase normalized to α-tub (from blots pictured in F, G). J, K. Quantification of total MOG normalized to α-tub (from blots pictured in F, G). L, M. Quantification of total MBP normalized to α-tub (from blots pictured in F, G). C, D: n = 4; H-M: n = 6; all comparisons non-significant by two-tailed Student’s t-test. C, D: data represent mean ± SD. H-M: data represent mean ± SEM. E: n = 100; data represent individual measurements, comparison non-significant by linear regression analysis. A: scale bar = 50 μm. B: scale bar = 2 μm.

No obvious size difference or myelination defects could be observed on examination of toluidine blue-stained optic nerves (Fig 6A). A more detailed analysis was performed in optic nerves by transmission electron microscopy (TEM; Fig 6B). WT and *Dst*^*dt-27J*^ optic nerves contained similar numbers of myelinated axons (Fig 6C). Further assessment of myelin sheath thickness by TEM revealed no overall difference in g-ratio between WT and *Dst*^*dt-27J*^ optic nerves (Fig 6D), nor was there any shift in myelin distribution based on axon caliber (Fig 6E). Finally, assessment of myelination status was done outside of the optic nerve by quantifying CNPase, MOG and MBP levels in both cerebral cortex and spinal cord, also revealing no change in any of these markers of mature myelin between WT and *Dst*^*dt-27J*^ (Fig 6F–6M).

## Discussion

The loss of functional neuronal Dst isoforms results in a severe phenotype characterized by ataxia, dystonia and death [21,22]. While the phenotype is largely a consequence of sensory neuron degeneration, previous work has implicated myelinating cells in both the PNS and CNS as contributors to, if not instigators of, the *Dst*^*dt*^ phenotype [23,24]. Specifically in the CNS, it has been argued that minor myelination pathologies are in fact early signs of OL-intrinsic defects in the CNS, and that further deterioration would occur if longevity of the animal model was not so limited [24]. However, tools to investigate this hypothesis were wanting at the time. With our present study, we sought to clarify the effects of Dst loss on OLs using the severe *Dst*^*dt-27J*^ model lacking all neuronal Dst isoforms. By employing methods to isolate and culture *Dst*^*dt-27J*^ OPCs and OLs *in vitro*, we were able to divorce OLs from not only the short phenotypic lifespan of the animal model but also from the influence of *Dst*^*dt-27J*^ neurons. This allowed us to observe OL-intrinsic behaviors in the absence of neuronal Dst.

Our results suggest that even lacking all three neuronal Dst isoforms, no intrinsic defects exist in OL differentiation at the morphological or molecular levels. This apparent regularity in OL behavior was not limited to a subpopulation of surviving healthy cells, as apoptosis was not increased in *Dst*^*dt-27J*^. Further, *in vivo* analysis revealed no deficits in CNS myelination of axons or in myelin sheath thickness in optic nerve, and no loss of CNPase, MOG or MBP in spinal cord or cerebral cortex. These findings are dissimilar to what was previously found in the less severe *Dst*^*dt-Tg4*^ model, which retains functional Dst-A3 [24]. While our results do not disqualify the existence of myelination defects in that model, it seems intuitive that if CNS myelination defects are indeed part of *Dst*^*dt*^ pathology then the more severe model would exhibit more severe CNS myelination problems. It must also be noted that while some qualitative *in vitro* analyses of OLs was undertaken in the initial study (revealing no obvious morphological defects in *Dst*^*dt-Tg4*^ OLs) [24], the tools to isolate and culture primary mouse OLs were limited and thus restricted the ability for a more comprehensive analysis of intrinsic OL biology, as we have done here.

We must also acknowledge that while this study provides fairly detailed molecular and morphological analyses, these assessments were done at a single pre-myelinating time point and at a point where terminal differentiation is known to occur for these cells *in vitro*. Thus, it is possible that differences in morphological and/or molecular development may occur at time points prior to terminal differentiation, which we did not investigate. However, since terminal differentiation is achieved with equal success in both WT and *Dst*^*dt-27J*^ OLs *in vitro* within the expected time line, any differences in their development prior to this point must be transient and are unlikely to affect their biological function as mature OLs. This is supported by our *in vivo* data, which reveal no defects in optic nerve myelination, cerebral cortex myelin protein expression or spinal cord myelin protein expression even at end-stage of the *dystonia musculorum* disease. Again, differences in developmental timing in OL maturation and myelination prior to this point cannot be ruled out *in vivo*, but if they do occur these too must be temporary as even end-stage *Dst*^*dt-27J*^ animals exhibit myelin status and health that is indistinguishable from WT animals. While our study is primarily concerned with the biological relevance of the loss of Dst in OPCs and OLs, additional investigation would need to be undertaken to clarify whether any transient differences in differentiation dynamics arise when Dst is absent.

Interestingly, intrinsic differentiation and myelination defects do exist in Schwann cells in both the *Dst*^*dt-Tg4*^ and *Dst*^*dt-27J*^ models, and are more severe when all three neuronal isoforms are absent [23]. This was illustrated in *in vitro* primary cultures and *in vivo* transplant experiments in which *Dst*^*dt-tg4*^ and *Dst*^*dt-27J*^ Schwann cells failed to differentiate or myelinate normally. While many similarities exist between Schwann cells and OLs—most obviously their function as producers of myelin—there are also many differences that may account for their apparent disparate requirement for neuronal Dst. First, intermediate filaments are present in Schwann cells and play a role in peripheral myelination, while OLs do not contain intermediate filaments [2,6,7,32]. Dst can bind some intermediate filaments in addition to microfilaments and microtubules, and loss of Dst leads to disorganization of intermediate filaments α-internexin, neurofilament and peripherin in sensory neurons [33,34]. A second key difference between OLs and Schwann cells is the production of a basement membrane. Following contact with an axon, Schwann cells surround their exterior layer with a basement membrane, the mechanical integrity of which is required for continued wrapping of the axon and subsequent myelin production [35,36]. Further, OLs and Schwann cells express a different complement of integrins, transmembrane heterodimeric complexes that convey signals between the extracellular matrix and intracellular space largely to elicit cytoskeletal reorganization [11,37,38]. It has been speculated that Dst may interact with some integrins to mediate cytoskeletal organization [23,24]. Thus it is possible that in Schwann cells, Dst is involved in any or all of intermediate filament organization, basement membrane formation and mediation of signaling of specific integrin complexes—none of which are present in OLs.

Further explanation may exist for the seemingly disposable nature of neuronal Dst in OLs. In mammals, a second member of the spectraplakin exists: microtubule-actin crosslinking factor 1 (Macf1; variantly actin crosslinking family 7/Acf7). Two isoforms of Macf1 are preferentially expressed in CNS tissues with little expression in PNS tissues, and share significant homology with neuronal Dst [39,40]. Macf1 can regulate cytoskeletal dynamics, and has both actin-binding and microtubule-binding domains, similar to Dst [40–42]. This raises the possibility that neuronal Dst does serve a function in the CNS, which may be compensated for by Macf1 if the former is lost. However, this idea was previously investigated in the *Dst*^*dt-27J*^ model; no increase in Macf1 was observed in brain or spinal cord from *Dst*^*dt*^ animals, as would be expected if additional Macf1 were required to fill a role normally played by Dst [40]. While this does not preclude a compensatory function for Macf1, it does suggest that—much like OLs and Schwann cells—Macf1 and neuronal Dst serve similar functions that are likely restricted to their respective nervous system compartments.

Finally, though the lack of Dst does not appear to be biologically relevant to the terminal differentiation of OLs during developmental myelination, it may be required for remyelination following a demyelinating injury. In the healthy CNS, any damage to mature myelin that may occur can be repaired by the differentiation of resident OPCs into new myelinating OLs, which subsequently ensheathe denuded areas of an axon. Though this process is believed to largely mimic the processes of developmental myelination—the so-called recapitulation hypothesis [43,44]–the final product of remyelination includes both thinner sheaths and shorter internodes than expected. This raises the possibility that different mechanisms are involved in remyelination than in developmental myelination, and this remains an important open question in OL biology. Interestingly, it has also been noted that in some instances of CNS demyelination, remyelination is carried out not by OLs but by Schwann cells[45]. These Schwann cells are not emigrated from the PNS, but surprisingly arise from CNS glial progenitors in specific circumstances when the injury results not only in demyelination but also in depletion of astrocytes at the injury cite [45,46]. The expression of dystonin has not been investigated in the context of remyelination. Future work should include the use of remyelination models applied to *Dst*^*dt*^ animals—such as organotypic brain slice culture—to explore the role of neuronal Dst in this important process, particularly when Schwann cells are implicated in CNS recovery from demyelination.

## Conclusions

Here, comprehensive *in vitro* and *in vivo* analyses were performed to better understand the role of neuronal Dst in OLs using the *Dst*^*dt-27J*^ animal model. Our results revealed that even in the absence of all three isoforms of Dst, OLs are able to differentiate normally both morphologically and molecularly. Survival of OLs lacking Dst is not compromised, nor is migration of OPCs. *In vivo* assessment of optic nerve myelination at disease end-stage showed normal density of myelinated axons and myelin thickness in optic nerve, as well as normal expression levels of CNPase, MOG and MBP in both spinal cord and cerebral cortex in animals with *dystonia muscolorum*. While we cannot rule out the occurrence of transient differences in OL maturity or that other cellular processes may be affected in OPCs and OLs in the absence of Dst, these data suggest that neuronal Dst is not essential for proper terminal OL differentiation or functional myelin development in the CNS.

## Supporting Information

S1 Fig Proliferating OPCs and differentiating OLs express neuronal *Dst* transcripts. Whole-gel view of RT-PCR *Dst*-*A1*, *-A2* and *-A3* with *actb* loading control in primary proliferating OPCs and differentiating OLs. L = ladder; bp = base pairs.
(TIF)

S2 Fig CNPase, MOG, and MBP expression is unchanged in *Dst*^*dt-27J*^ cerebral cortex and spinal cord. Whole-membrane view of all CNPase, MOG, and MBP isoforms, as well as α-tubulin (green) in cerebral cortex (CC) and spinal cord (SC) from P15 wild-type (WT) and *Dst*^*dt-27J*^ mice. L = ladder; kDa = kilodaltons.
(TIF)
