# Supplementary figures and images for: Cytoskeletal Linker Protein Dystonin Is Not Critical to Terminal Oligodendrocyte Differentiation or CNS Myelination
Source: PLoS One. 2016 Feb 17;11(2):e0149201. doi: 10.1371/journal.pone.0149201 (PMC4757544; doi:10.1371/journal.pone.0149201)

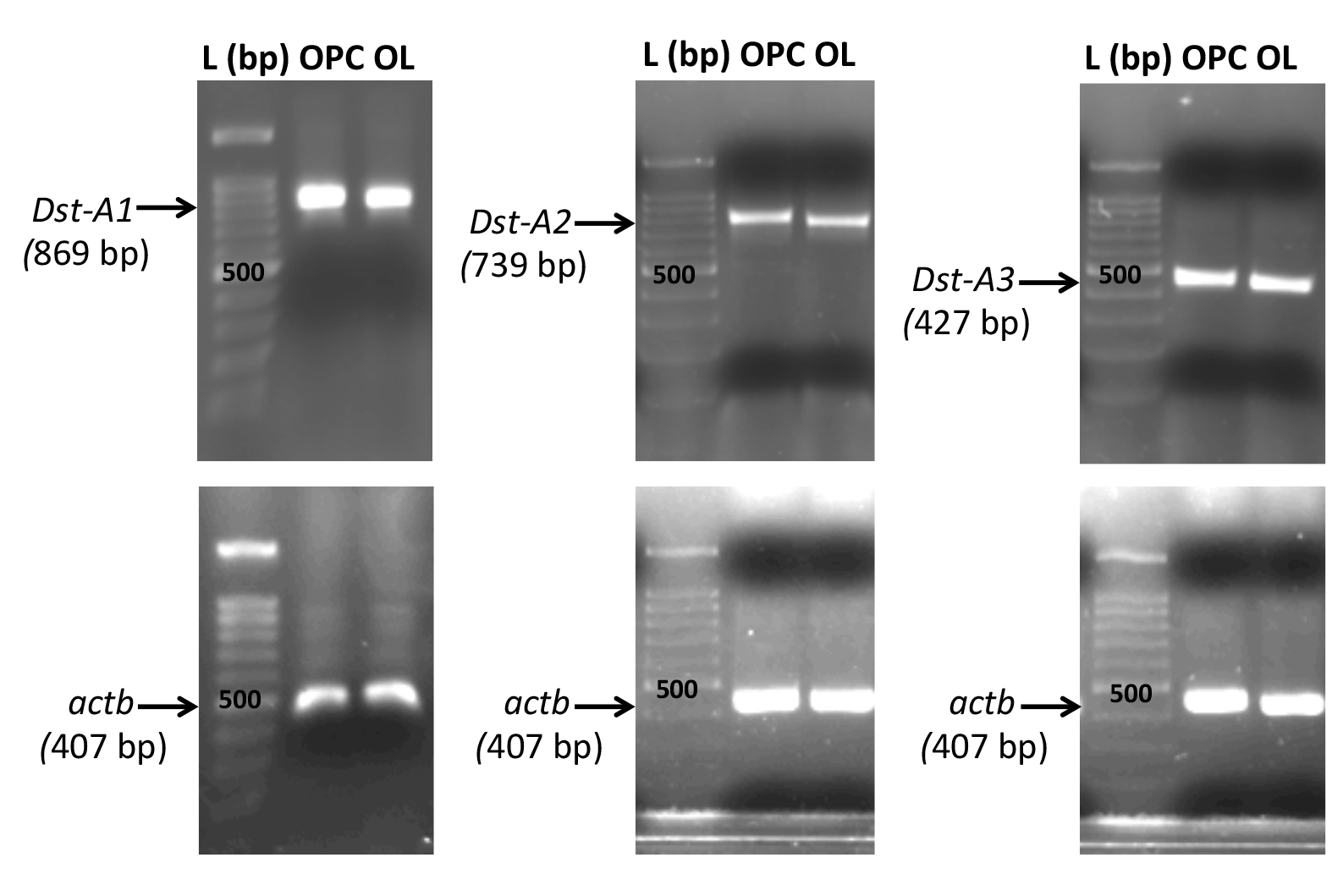

Supplement: S1 Fig — Whole-gel view of RT-PCR Dst-A1, -A2 and -A3 with actb loading control in primary proliferating OPCs and differentiating OLs. L = ladder; bp = base pairs. (TIF) [file pone.0149201.s001.tif]

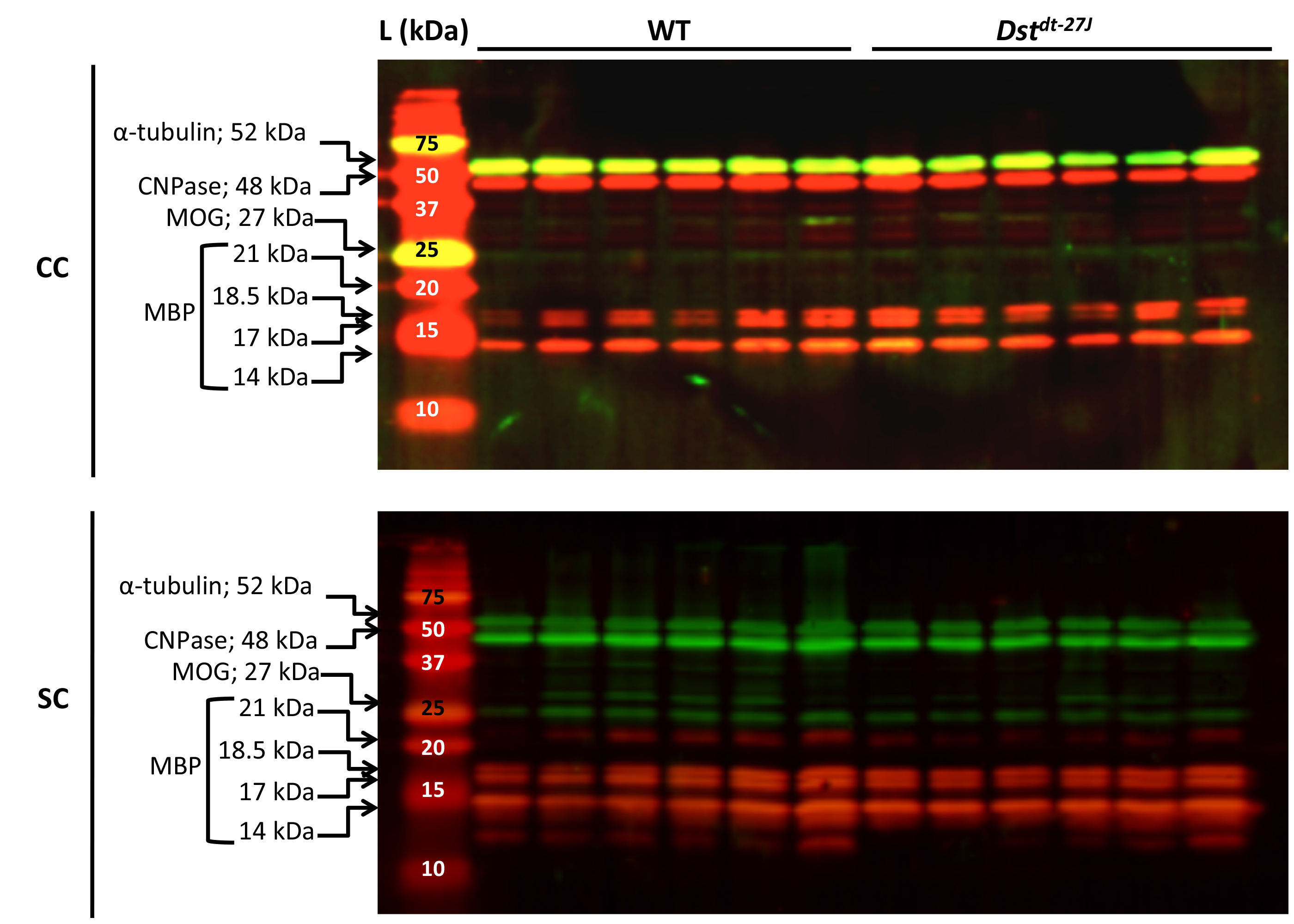

Supplement: S2 Fig — Whole-membrane view of all CNPase, MOG, and MBP isoforms, as well as α-tubulin (green) in cerebral cortex (CC) and spinal cord (SC) from P15 wild-type (WT) and Dstdt-27J mice. L = ladder; kDa = kilodaltons. (TIF) [file pone.0149201.s002.tif]
